# Laryngeal Electromyography in the Therapeutic Process of Patients with Vocal Fold Immobility or Dysmobility

**DOI:** 10.3390/life12030390

**Published:** 2022-03-08

**Authors:** Paulina Krasnodębska, Agata Szkiełkowska, Ludmiła Czarkwiani-Woźniakowska, Beata Miaśkiewicz, Anna Sinkiewicz, Henryk Skarżyński

**Affiliations:** 1 Audiology and Phoniatrics Clinic, Institute of Physiology and Pathology of Hearing, Mochnackiego 10, 02-042 Warsaw, Poland; p.krasnodebska@ifps.org.pl (P.K.); a.szkielkowska@ifps.org.pl (A.S.); 2 Department of Neurosurgery, The Children’s Memorial Health Institute, Av. Dzieci Polskich 20, 04-730 Warsaw, Poland; ludmila23@wp.pl; 3 Department of Otolaryngology and Phoniatry and Audiology, Collegium Medicum, Nicolaus Copernicus University, 87-100 Torun, Poland; anna.sinkiewicz@cm.umk.pl; 4 Otorhinolaryngologic Clinic, Institute of Physiology and Pathology of Hearing, Mochnackiego 10, 02-042 Warsaw, Poland; h.skarzynski@ifps.org.pl

**Keywords:** laryngeal electromyography, vocal fold, laryngeal paralysis, vocal fold dysmobility

## Abstract

(1) Background: Laryngeal electromyography (LEMG) plays a key role in classifying the severity of nerve damage and determining the prognosis of the nerve recovery. LEMG is primarily a qualitative study, without a standardized approach to interpretation. The development of qualitative and quantitative analysis would situate LEMG in the gold standard of modern neurolaryngologic diagnostics. The aim of this study was to quantitatively evaluate laryngeal electromyography recorded in patients with vocal fold immobility or dysmobility. (2) Methods: The electromyographic material comprised 84 thyroarytenoid muscles recordings of 42 patients. (3) Results: In our study, we observed significant differences between EMG characteristics of healthy and paralyzed VF. Our study showed that recording laryngeal muscle activity during successive phases of breathing provides additional valuable information. We noticed that the frequency and amplitude of motor unit potentials correlates with the return of vocal fold functionality. (4) Conclusions: Laryngeal EMG guides the clinician on the best course of treatment for the patient. It is therefore important to develop an effective methodology and consensus on the quantitative interpretation of the record. Amplitude and frequency parameters are valuable in predicting neural recovery and in the return of vocal fold mobility.

## 1. Introduction

Laryngeal electromyography (LEMG) plays a key role in classifying the severity of nerve damage and determining the prognosis of the nerve recovery [1]. However, according to the literature, most controversy surrounds the prognostic accuracy of LEMG [2]. Rickert et al. [3] concluded in a meta-analysis that LEMG is a good predictor of poor recovery but a much less reliable predictor of good recovery. In the interpretation of EMG of other muscles, the Seddon classification is used to assess the degree of damage and prognosis [4]. In studies of the larynx, near normal voluntary motor unit potentials and recruitment without spontaneous activity, according to the Seddon classification, are indicative of neuropraxia, which may have a good prognosis. In contrast, the absence of voluntary motor units and spontaneous activities, such as fibrillation potentials, reflects axonotmesis or neurotmesis, which indicates a poor prognosis for laryngeal recovery [5]. Prognosis using Seddon’s classification in laryngeal examination is limited because the used prognostic criteria vary from study to study. Furthermore, some authors suggest that Seddon’s characterization principles may not fully correspond to the larynx, due to the low number and size of fibers per motor unit (MU) within the laryngeal muscles [2,6]. The importance of LEMG and its impact on clinical practice has resulted in extensive work on standardization of the test methodology. The ability to predict the course of laryngeal reinnervation allows for earlier decisions on the most appropriate therapy, including early surgical intervention [7]. If LEMG cannot be performed, at least 9 months of observation of the paralyzed larynx is recommended, as the process of laryngeal nerve regeneration takes approximately one year. Such a long waiting period is particularly burdensome for the patient.

Laryngeal electromyography is primarily a qualitative study, without a standardized approach to interpretation [8,9]. For several years, leading otolaryngological research units have been working to introduce parametric evaluation of LEMG. The development of qualitative and quantitative analysis would situate the LEMG in the gold standard of modern neurolaryngologic diagnostics.

The aim of this study was to quantitatively evaluate laryngeal electromyography recorded in patients with vocal fold immobility or dysmobility. Our aim was to determine which elements of the amplitude and frequency parameters of LEMG are most valuable in predicting neuronal recovery and the return of vocal fold mobility.

## 2. Material

**Figure 1:**
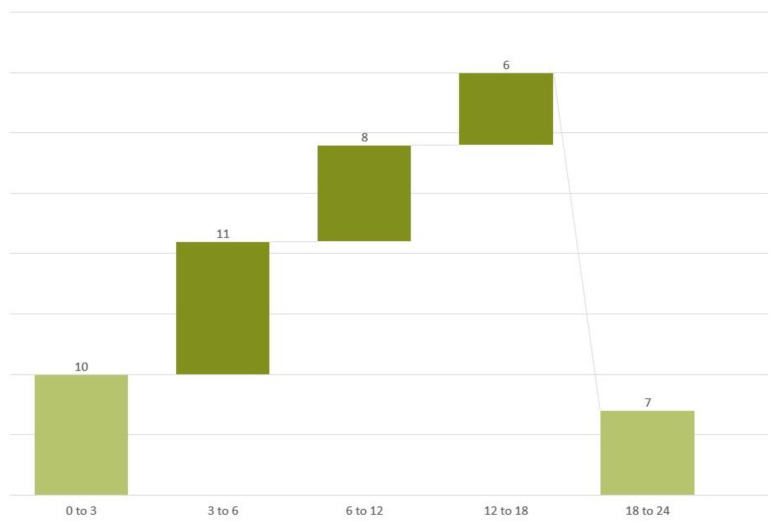
Patients divided into groups according to the occurrence of vocal folds (VF) paralysis.

The material of this study consisted of 42 patients with laryngeal dysmobility hospitalized in the Department of Audiology and Phoniatrics in 2018. These patients were referred to our clinic for voice therapy due to dysphonia in the course of unilateral laryngeal paralysis. There were 23 women and 19 men with a mean age of 56 years (standard deviation (SD): 14). Figure 1 shows the patients divided into groups according to the presence of vocal fold (VF) paralysis. 

Only patients who were admitted between 3 months and 2 years after the onset of dysphonia due to unilateral laryngeal paralysis were included in the study. The main causes of the paralysis were thyroid surgery (18 patients) and idiopathic cause (12 patients). Other surgical procedures in the neck or chest or trauma to this area caused laryngeal paralysis in the remaining 12 patients. 

The study was positively evaluated by the bioethics committee KB.IFPS 1/2021.

## 3. Methods

An acoustic voice analysis was performed in each patient. The patient’s voice was recorded during prolonged phonation of the vowel [a] at a convenient pitch and volume. Spectrograms were scored according to the 5-point Yanagihara scale (0 = normal, 4 = severely abnormal) [10].

### 3.1. Laryngeal Endoscopy

Vocal fold immobility or dysmobility was diagnosed by indirect videolaryngoscopy using a rigid or flexible endoscope. Laryngovideostroboscopic (LVS) features were assessed during phonation when the patient phonated the vowel [a] with a modal pitch and regular loudness. Patients were followed up for one year. Paralysis was diagnosed—if there were no visible movements or they were passive. Paresis—if the patient presented limited vocal fold movements. We confirmed the neurological etiology of vocal fold dysmobility by LEMG [11] examination. We divided the group of patients with vocal fold paresis into two subgroups: substantial mobility with reduced but functional movement and minimal mobility with absent functionality of the vocal fold. We described free mobility if appropriate vocal fold function was observed during respiratory and phonatory tasks [1]. 

### 3.2. LEMG Recordings

The electromyographic material comprised 84 thyroarytenoid muscles (TA)-EMG recordings. LEMG was performed by a physician, phoniatrist certified in electrodiagnostic medicine and analyzed together with neurologist, clinical neurophysiologist certified in electrodiagnostic medicine. LEMG signals were obtained using a 45 × 0.45 mm concentric needle electrode on a Neurosoft EMG device. A surface disk ground electrode was adhered to the patient’s forehead. Our protocol for the LEMG has been published previously [12]. Both VFs were examined in each patient. The non-paralyzed VF was examined first. For the study, recordings from the TA muscles during sustained phonation of the vowel [a] and during breathing phases were analyzed. The examination was performed in a sitting position, with the patient’s neck slightly extended and the head steadily resting on the back of the chair. TA muscle was examined with a needle electrode transcutaneously, after cleansing the skin with alcohol. The concentric electrode was inserted in or slightly lateral to the midline of the neck (2 to 5 mm left or right) at the level of the cricothyroid ligament. The electrode was directed laterally and superiorly towards the upper horn of the thyroid cartilage (at 45° upwards, outwards, and backwards). An increase in electrical activity during phonation confirmed the correct position of the electrode. The patient was asked to steadily produce three series of [a]s of moderate intensity. We asked patients to choose their own comfortable pitch. 

The LEMG signal from the tested muscle was analyzed and categorized according to the standardized LEMG protocol for intentional activity into normal, diminished, greatly diminished, or absent recruitment [13,14]. Normal MU recruitment was that with an absence of fibrillation or positive wave potentials. Diminished MU recruitment was repetitive firing of motor units and a reduced interference pattern with normal or polyphasic MU and possible fibrillation or positive waves but not complex repetitive discharges during spontaneous activity. Absent or greatly diminished MU recruitment was a reduced interference pattern with normal or polyphasic MU and possible fibrillation, positive waves, or complex repetitive discharges during spontaneous activity [2]. Synkinesis was suspected in patients with detectable MUP activation in agonistic and antagonistic TA muscle maneuvers during vocalization and breathing. The recruitment pattern of the impaired VF was compared with that of the healthy fold (with normal mobility). We analyzed further quantitative parameters according to LEMG: MUP frequency and amplitude. Following Wang’s results, we determined the quantitative parameters of the impaired TA muscle of the vocal fold from >80% as normal to almost normal [5].

### 3.3. Statistical Analysis

The following tests were used to statistically analyze the parameters obtained in this study (time from onset of paralysis, degree of paralysis, quantitative LEMG scores): Pearson correlation and Mann–Whitney test. The level of statistical significance was set at *p* < 0.05.

## 4. Results

On admission, LVS showed VF paralysis in 29 patients and paresis in 13 patients (of which seven patients had reduced but functional movement and six patients had minimal mobility with absent functionality). The acoustic voice analysis showed a mean value of 2.55 degrees of hoarseness according to Yanagihara (SD: 1). After 1 year of follow-up, 27 VFs remained paralytic.

### 4.1. Muscle Activation during Phonation

**Figure 2:**
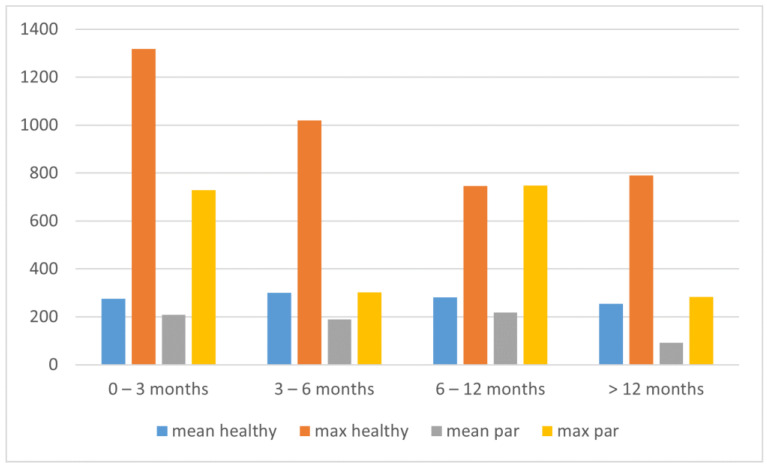
Mean and maximum amplitudes of the healthy and paralyzed/paresis [denoted as ‘par’] VFs divided by the time of paralysis occurrence.

Patients underwent a standardized LEMG protocol consisting of a qualitative EMG waveform assessment. In 14 patients, we observed normal or near normal motor unit recruitment. In 17 patients, we found fibrillations or positive sharp waves indicating ongoing denervation of muscle fibers. In 12 patients, no muscle activity was recorded (mean and maximum amplitude ranged from 0–120). Figure 2 presents the mean and maximum amplitudes of healthy and dysmobile VFs according to the time of onset of the paralysis. 

In healthy VFs, the mean amplitude of the MU action potential from the TA muscle was 328 μV (SD 231), and the maximum was 1388 μV (SD 1112). In larynxes with unilateral paralysis not older than six months, a significantly increased maximum amplitude of healthy VFs was observed. A trend of decreasing maximum amplitude of healthy VFs was seen, which may have been due to irritation of the TA muscle nerve fibers, but which did not manifest clinically and did not affect the mobility assessed in LVS. The elevated amplitude normalized after approximately 6 months. A decrease in mean amplitude in paralyzed VFs at 12 months after paralysis was also evident. The mean values decreased from 205 μV to 91 μV at 1 year. A statistically significant difference was found between the mean amplitude of paralyzed VFs immobilized for no more than 12 months and for more than 12 months.

### 4.2. Muscle Activation during Breathing

Our methodology involved measuring muscle electrical activity at rest and during voluntary recruitment. Higher values of amplitudes during inspiration than expiration was observed in both healthy and paralyzed folds. The medians of the mean and maximum amplitudes during inspiration in healthy VFs were 50 μV and 183 μV, while in paralyzed VFs, they were 0 μV and 100 μV. The mean values were, respectively, 94 μV (SD: 90) and 263 μV (SD: 238), and on the paralyzed side, they were 52 μV (SD: 78) and 169 μV (SD: 157). The medians of the mean and maximum amplitudes during expiration of the healthy VFs were 80 μV and 215 μV, while those of the paralyzed VFs were 0 μV and 53 μV. The mean values were, respectively, 95 μV (SD: 90) and 303 μV (SD: 386), and on the paralyzed side, they were 35 μV (SD: 58) and 95 μV (SD: 81). Eight patients showed features of abnormal TA stimulation during the inspiratory phase. In these patients, an abnormally increased amplitude was observed in the paralyzed folds compared to the healthy side during inspiration. We suspected synkinesis of the TA muscle in these patients. In four patients from this group, we observed partial recovery of the paralyzed fold after 1 year, but the voice quality of these patients was unsatisfactory due to glottal insufficiency.

### 4.3. VF Recovery

A one-year follow-up after unilateral laryngeal paralysis showed restoration of free mobility of the paralyzed VFs in four patients. Nine patients regained substantial mobility, eight regained minimal mobility, and 21 VFs remained immobile. Observations of VF mobility and spectrogram results at the one-year follow-up are shown in Table 1. 

**Figure 3:**
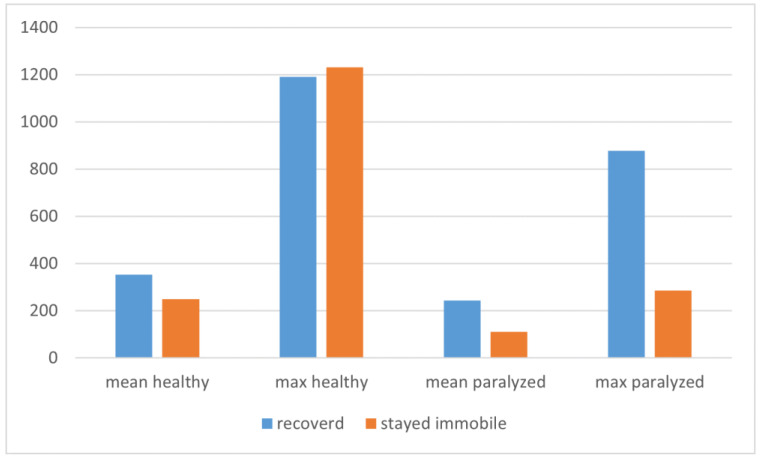
Mean and maximum amplitude of healthy and paralyzed vocal folds according to the course of recovery (mobile/immobile).

During this period, we observed a return of VF mobility (partial or complete) in 30% of patients (3 of 10) who were admitted to our clinic 3 months after dysphonia onset, in 19% (4 of 21) who were admitted 6 months after dysphonia onset, in 27% (8 of 29) who were admitted 1 year after dysphonia onset, and in 0% (0 of 13) of patients with paralysis older than 1 year. In addition, we observed the best voice results assessed in the spectrogram in patients who were referred to our clinic for voice therapy within the first 6 months of symptom onset. Figure 3 shows the mean and maximum amplitude of healthy and paralyzed vocal folds according to the course of recovery (mobile/immobile).

The statistical analysis showed no differences between the healthy vocal folds of patients in both groups. It was found that the mean and maximum amplitude during volitional activity was significantly higher in the paralyzed vocal folds of patients who recovered vocal fold mobility. In contrast, the absence of volitional motor units and spontaneous activity indicated a poor prognosis for laryngeal recovery. In 12 patients, no muscle activity was recorded during phonation (mean and maximum amplitude ranged from 0–120). In this group, we observed a partial improvement of VF in one patient. We divided the rest of the group into two subgroups: patients in whom VF movement was restored and patients in whom VF movement was not restored. The mean and maximum amplitudes in the groups were 242 μV (SD: 175 μV) and 798 μV (SD: 777 μV), and in the second group of patients who did not recover, they were 158 μV (SD: 73 μV) and 465 μV (SD: 363 μV). The trend of significantly higher amplitude values was still evident in patients who recovered VF mobility. Significantly higher values of maximum amplitudes were recorded among patients who presented MU activity during phonation and who recovered VF mobility. 

**Figure 4:**
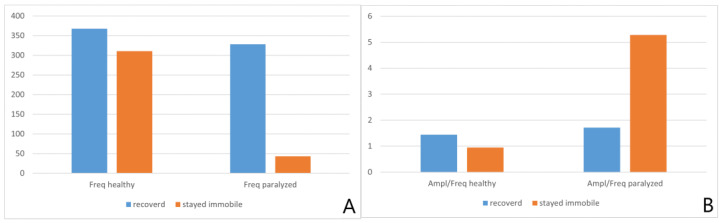
Values of frequency (**A**) and the amplitude-to-frequency ratio (**B**), depending on the examined VF and mobility return.

A further parametric analysis of the EMG recordings involved observing differences in the frequency of MU discharges, which is an objective indicator of an interference pattern. The increased discharge frequency of the paralyzed VF compared with the healthy one was a positive prognostic feature of EMG (Figure 4A). In contrast, a high amplitude-to-frequency ratio was an unfavorable feature (Figure 4B). These differences were statistically significant.

## 5. Discussion

The material of the present study showed that iatrogenic causes are still the most common cause of vocal fold paralysis. Our observations are consistent with epidemiological data presented by other authors [15,16].

Laryngeal electromyography is a unique test among EMG. Its interpretation requires the combined skills of a neurologist, otolaryngologist, and phoniatrist. The methodology of the examination is still being developed and improved. Munin concluded that methodological differences found in previous studies limit the usefulness of LEMG. The main problems noted by the author related to heterogeneous testing techniques, different definitions of vocal fold paralysis, and inconsistent determination of prognostic features based on LEMG. Another reason may be the different time of examination from the onset of paralysis. Furthermore, according to Stanisz [17], the different timing of LEMG after the diagnosis of vocal fold paralysis may cause differences in the reporting of the incidence of laryngeal synkinesis. As our results show, the discrepancy in patient reporting is still large.

### 5.1. Qualitative Assessment

Qualitative EMG MUP characterization is the accepted standard for describing LEMG. It consists of assessment of the configuration and recruitment of motor units, detection of fibrillation, and the presence of synkinesis. However, due to the inadequate characterization of laryngeal pathology based on qualitative assessment alone, scientific societies worldwide are encouraging further work on LEMG guidelines, including quantitative description methods. 

In our study, we observed significant differences between the EMG characteristics of healthy and paralyzed VFs. Examining paralyzed VFs, we observed near-normal voluntary motor unit potentials and recruitment without spontaneous activity in 33% of patients, absent voluntary motor units in 28% of patients, and spontaneous activity in 40% of patients. Similarly, Foerster [15] observed some signs of pathological reinnervation in the LEMG in the vast majority of patients studied. Lin observed a normal MU configuration in 44% of patients. Moreover, he registered adductors synkinesis in 30% of patients [16]. 

Our study showed that recording laryngeal muscle activity during successive phases of breathing provides additional valuable information. Characteristic features of muscle activation may indicate ongoing synkinesis. Paniello [7] observed increased TA muscle amplitude during respiratory activity more frequently in cases of synkinesis than in the control group. Foerster [15] stated that TA muscle synkinesis is rarely observed in good prognosis. We recorded abnormal activation during breathing in 19% of patients. Among the patients, we noticed a higher percentage of return of vocal fold mobility (50%) than in the rest of the population. However, the quality of return was unsatisfactory in all these patients, characterized by incomplete return of mobility and glottal phonatory insufficiency.

### 5.2. Quantitative Description

According to a meta-analysis by Rickert et al. [3], most studies do not define the near-normal state quantitatively. Ha [8] evaluated the differences between normal and paralyzed vocal folds and the cut-off values of MU amplitude. He observed that a paralyzed vocal fold had a significantly lower amplitude than a normal one. The MUAP amplitude cut-off value for unilateral VFP was 68.35 µV [8]. Heman-Ackah [18] noted that the MUP usually has an amplitude of 200–500 µV. Our results showed significant differences in the amplitude range between the healthy and paralyzed VFs, as well as different recording characteristics in patients with present synkinesis. According to Hillel [13], the quantitative analysis based on amplitude observation during phonation tasks is indicative of the condition of the efferent motor nerve and is diagnostic for denervation, reinnervation, or a synkinetic process.

### 5.3. Prognostics

#### 5.3.1. Based on Qualitative Assessment

Munin [19] described an evidence-based consensus statement on the use of LEMG in the diagnosis and treatment of vocal fold paralysis after recurrent laryngeal neuropathy based on a systematic review using the American Academy of Neurology criteria for the rating of diagnostic accuracy. The author described that active voluntary recruitment of motor unit potentials and the presence of polyphasic motor unit potentials during the first 6 months after lesion onset predict recovery. Furthermore, Smith [20] described an excellent prognosis for the recovery of vocal fold movement when there is good to normal motor recruitment, no signs of denervation, and no signs of synkinetic activity using LEMG. Munin stated that the occurrence of positive sharp waves and/or fibrillation potentials did not affect prognosis [19]. Furthermore, he observed a polyphasic MU more frequently in patients who recovered VF activity [2]. Munin found that a qualitative LEMG analysis based on motor unit recruitment, interference pattern, and spontaneous activity was able to predict sustained UVFP with a good or poor LEMG signal in 91% of patients. Hillel [13] confirmed in his study that recruitment of motor units during a phonation task is the element most predictive of the outcome. Sittel [1] predicted the laryngeal paralysis outcome based on Seddons classification. In axonotmesis (a spontaneous activity indicative of neuronal degeneration), the predictive value for a poor outcome was 94.4%. A favorable outcome in neuropraxia (a diffuse recruitment pattern or single action potential with voluntary activity without fibrillatory activity or positive sharp waves) was accurate in 12.8% [1].

Many authors describe the recovery of muscle innervation in up to 80% of paralyzed VFs, which is not synonymous with the return of VF mobility [15,20]. In our material, we observed a recovery of muscle innervation in 71% of patients. In our material, we observed a partial return of VF motion in 41% of patients and complete recovery of function in 9%. Our observations are similar to those found in the literature. According to Sittel’s [1] observations, 45% of paralyzed vocal folds remain immobile. The author observed return of fold mobility in 55% of patients, of which only 8.1% regained completely free mobility of the paralyzed fold. Literature results and our own experience show that the return of function does not depend solely on the presence of reinnervation. The regeneration of nerve fibers in laryngeal muscles is more frequent than was thought in the past. The low percentage of complete return of VF mobility is influenced by the presence of synkinesis. As stated by Munin [19], the presence of electrical synkinesis may decrease the likelihood of recovery. Foerster [15] further observed that evaluation of the posterior cricoarythenoid (PCA) muscle increased the likelihood of prognosis. They observed that 35.7% of paralyzed vocal folds had normal or near normal TA innervation, whereas the corresponding PCA tracings were pathological in 94.6%. We observed features of abnormal TA excitation during respiration, which may be an indicator of synkinesis, pointing in the direction of the reinnervation process when the PCA test could not be performed.

#### 5.3.2. Based on Quantitative Assessment

In our work, we noticed that the frequency and amplitude of recording correlated with the return of vocal fold functionality. Similar observations relating to the parameter turn analysis were described by Smith [20]. A quantitative analysis of frequency parameters and turn analysis has often been found as the best prognostic parameter [20,21]. 

The results of our study showed significantly higher amplitude values in patients who recovered VF mobility. Moreover, we observed an increased discharge frequency of the paralyzed VF as a positive prognostic feature and a high amplitude-to-frequency ratio as an unfavorable feature.

In deciding on early intervention, the features of synkinesis found on EMG should be considered. Intra-pharyngeal muscle synkinesis associated with unilateral vocal fold paralysis (UVFP) is thought to preserve the tone of the thyroarytenoid-lateral cricoarytenoid muscle complex, resulting in improved voice despite the presence of vocal fold paralysis [2]. Furthermore, patients with unilateral vocal fold paralysis and LEMG evidence of laryngeal synkinesis are more likely to have a less noticeable voice impairment than those without synkinesis [22]. As our previous studies have shown, functional voice therapy, including phoniatric, logopaedic, and physiotherapeutic therapy, started as early as possible can provide the best possible voice results [23].

According to the literature, in cases of vocal fold mobility disorders, diagnostic needle EMG provides the most information to support management decisions and to identify candidates for early intervention [13,24]. Patients with reduced or absent MU recruitment are not capable of maintaining muscle tone due to progressive vocal fold muscle atrophy. Patients from this group will be candidates for surgical intervention to prevent incorrect phonation mechanisms as early as possible. 

The laryngeal nerve is expected to take approximately one year to regenerate, so it is recommended that people with laryngeal paralysis be observed before deciding on any surgical intervention. On the other hand, this long waiting period in uncertainty and the need to select an appropriate therapy have led to the widespread use of LEMG in the early prognosis of vocal fold paralysis. By knowing the pathophysiology, the clinician can make therapeutic choices based on an understanding of the etiology of the disorder [25,26]. As shown in our study, as early as 3 months after paralysis, we observed signs of return of TA muscle function in 14% of all patients in whom VF function returned. In another 5% (making a total of 19%), recovery was observed in up to six months after the onset of paralysis. In this group were all patients in whom we observed full recovery of VF. We did not observe a return in patients whose paralysis lasted longer than one year. There are few papers describing the course of reinnervation in humans. Authors report that in animal studies, it can be seen as early as after 3 months [7]. Half of the dogs that recovered mobility had a good-prognostic EMG by 3 months. The other half developed their positive prognostic EMG later.

LEMG predicts a poor prognosis better than a good one. Absent MU recruitment is associated with a 90% rate of permanent paralysis [3,27]. In the absence of features of reinnervation in LEMG and the patient’s expectation of improved voice quality, it is reasonable to apply laryngeal augmentation with hyaluronic acid as early as possible. In this context, it is apropos to quote from Wang’s publication [5] that “an accurate prediction of no recovery is more important than a prediction of recovery”. Our clinical experience suggests that patients with poor conservative treatment results who do not show reinnervation patterns in LEMG should be offered injection laryngoplasty [23]. Medialization with an absorbable injectable, such as hyaluronic acid, gives good voice quality results. The effect is reversible, allowing time for eventual nerve regeneration. Our experience shows that, depending on the area of application, a different persistence of effects is obtained [28]. Our experience is in line with the approach of other authors, who encouraged patients with a poor prognosis to undergo permanent laryngeal skeletal surgery, such as type I thyroidectomy or arytenoid adduction, once the injected hyaluronic acid was absorbed [5]. Furthermore, as reported by Belsky [29], the presence of augmentation material in the vocal fold (after injection laryngoplasty) did not interfere with the LEMG recording. It was still possible to observe predictors of the return of vocal fold motion in the described patients with acute and subacute vocal fold paralysis.

Laryngeal EMG guides the clinician on the best course of treatment for the patient. It is therefore important to develop an effective methodology and consensus on the quantitative interpretation of the record. The authors’ quantitative analysis of the electromyographic recording demonstrated the usefulness of amplitude and frequency parameters. The paper shows that MUP analysis provides quantitative differences between normal and affected vocal folds. Moreover, amplitude and frequency parameters are valuable in predicting neural recovery and in the return of vocal fold mobility. The results of the study provide a useful contribution to the developing methodology of quantitative laryngeal EMG interpretation. Another important conclusion of the work is to highlight the importance of the methodology of electromyographic recording of the thyroarytenoid muscle. In the authors opinion, the examination should be recorded during phonation as well as during individual phases of breathing.

## Figures and Tables

**Table 1:** Observations of VF mobility and spectrogram results based on a one-year follow-up. Patients group divided according to the occurrence of unilateral laryngeal paralysis. Statistically significant difference between the results made during admission and follow-up are marked with * (*p* < 0.05).

Time from Paralysis In months	No of Patients	Visible Mobility of the VF		Spectrogram (Yanagihara Grade)	
		Admission to Clinic	1 year folow-up	Admission to Clinic	one-year folow-up
<3	10	2	5	2.4 (SD: 0.6)	1.9 (SD: 1) *
<6	21	4	8	2.7 (SD: 0.8)	2.2 (SD: 1.1) *
<12	29	7	15	2.7 (SD: 0.8)	2.4 (SD: 1)
12–24	13	6	6	2.1 (SD: 1)	2.1 (SD: 1)

## Data Availability

The data presented in this study are available on request from the corresponding author. The data are not publicly available due to protection of personal medical data.

## Abbreviations

The following abbreviations were used in this manuscript:

LEMG	Laryngeal electromyography
MU	motor unit
VF	vocal fold
LVS	Laryngovideostroboscopy
EMG	electromyography
TA	thyroarytenoid muscle
MUP	motor unit potential
PCA	posterior cricoarythenoid muscle
UVFP	unilateral vocal fold paralysis
